# Untreated Giant Macroprolactinoma with Chronic Cerebrospinal Fluid Leakage: An Unusual Complication

**DOI:** 10.1155/2019/4825357

**Published:** 2019-01-15

**Authors:** Mohamad Nazrulhisham Mad Naser, Nor Azizah Aziz, Noor Khairiah A. Karim

**Affiliations:** ^1^Department of Medicine, Hospital Pulau Pinang, Jalan Residensi, 10990 Georgetown, Pulau Pinang, Malaysia; ^2^Department of Cardiology, Hospital Pulau Pinang, Jalan Residensi, 10990 Georgetown, Pulau Pinang, Malaysia; ^3^Endocrinology Unit, Department of Medicine, Hospital Pulau Pinang, Jalan Residensi, 10990 Georgetown, Pulau Pinang, Malaysia; ^4^Regenerative Medicine Cluster, Advanced Medical and Dental Institute, Universiti Sains Malaysia, Bertam, 13200 Kepala Batas, Pulau Pinang, Malaysia

## Abstract

Macroprolactinoma has the potential to cause base of skull erosion and often extends into the sphenoid sinus. Rapid shrinkage of this invasive tumor following dopamine agonist therapy has been postulated to cause unplugging of the eroded area, leading to cerebrospinal fluid leakage. To the best of our knowledge, the occurrence of spontaneous cerebrospinal fluid leak in treatment-naive prolactinomas is very rare, the majority of which involve undiagnosed macroprolactinomas. We describe here a lady presented late with giant macroprolactinoma, complicated by cerebrospinal fluid leakage. This case raised the dilemma in the management pertaining to the role of either pharmacotherapy or surgical intervention, or combination of both. As she strictly refused surgery, she was treated with bromocriptine which was later changed to cabergoline. On follow-up, there was cessation of cerebrospinal fluid leak, marked reduction of serum prolactin level, and imaging evidence of tumor shrinkage. The majority of patients with medically induced cerebrospinal fluid leakage will require surgical procedures to overcome this complication; however, there are isolated cases of leakage resolution on continuing dopamine agonist therapy while awaiting surgery. The use of dopamine agonist does not necessarily cause worsening of cerebrospinal fluid leakage and instead may produce spontaneous resolution as in this case.

## 1. Introduction

The term giant adenoma was first described by Jefferson in 1940 for pituitary adenoma which has large dimensions, reaching maximum diameter of more than four centimeters with suprasellar extension [1]. Among all the pituitary tumors, prolactinoma appears to be unique as it has the potential to cause base of skull and sellar floor erosion which often extends into the sphenoid sinus and disrupts the dura. Rapid shrinkage of this invasive tumor following dopamine agonist (DA) therapy has been postulated to cause unplugging of the eroded area in the skull base and subsequently leading to cerebrospinal fluid (CSF) leakage [2–10]. Pituitary surgery and to a lesser degree radiotherapy are also recognized causes for CSF leakage in these patients [8, 9]. To the best of our knowledge, the occurrence of spontaneous CSF leak in treatment-naive prolactinomas is very rare and has only been reported in isolated cases or small series worldwide, the majority of which involve undiagnosed macroprolactinomas [4, 5]. We describe here a lady presented with late manifestation of giant macroprolactinoma, complicated further by spontaneous CSF leakage, raising dilemma in the management pertaining to the role of either pharmacotherapy or surgical intervention, or combination of both.

## 2. Case Presentation

A 32-year-old lady with normal cognitive function has presented with few symptoms and signs relating to pituitary gland disorder at different timeline but refused to seek early medical care. Firstly, she had primary amenorrhea which she initially thought may be a constitutional delay, but over time, she eventually came to term and decided not to get married or conceive to self-mitigate this problem. She then developed blurring of vision at the age of 19 years, but she just coped with it as she claimed the onset was insidious and she was still able to perform her routines. She had no significant headache and other signs to suggest increased intracranial pressure. At the age of 23, she started working as a factory operator but often experienced lethargy even on mild exertion, forcing her to take multiple sick leaves. She also noticed occasional spontaneous milky discharge from both nipples that stained her inner wear, but she dismissed this sign. As these problems progressed, she quitted her job and stayed at home with her parents. Three years later, she started to have dripping of clear fluid through her nose upon bending down and during strenuous activity. She eventually came forward for medical assistance as the latter symptoms really affected her daily activities.

On physical examination, she was normotensive. There were no signs of Cushing syndrome. Funduscopic examination revealed left optic atrophy secondary to compressive optic neuropathy, with left temporal hemianopia and almost right temporal hemianopia seen on visual acuity assessment. Hormonal assay investigations disclosed serum prolactin of 4200 mIU/L with dilutional assay of 250,688 mIU/L. There was reduced level of estradiol (62 pmol/L), follicular stimulating hormone (0.9 IU/L), and luteinizing hormone (0.1 IU/L). Thyroid function test showed normal thyroid stimulating hormone (1.78 mIU/L), low T4 (8.4 pmol/L), and normal T3 (4.3 pmol/L). Synacthen test revealed good cortisol response with baseline 0 hour of 394.4, 616.2 at half an hour and 785 at one hour. Her ‘chronic rhinorrhea' was proven to be CSF leak confirmed by clinical test (positive halo sign) and biochemical test (nasal cavity CSF glucose level of 3.9 mmol/L related to plasma glucose of 5.6 mmol/L).

**Figure 1:**
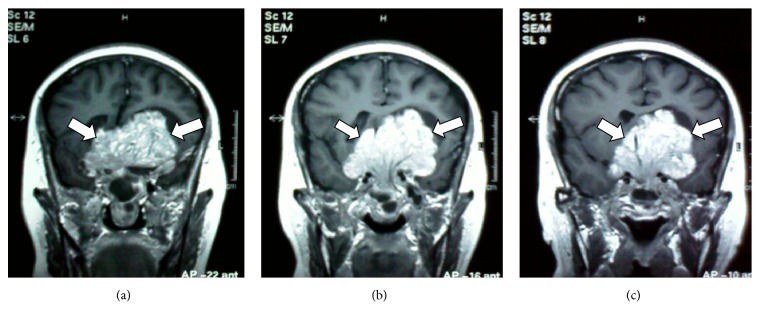
Initial contrast enhanced coronal T1-weighted sequence on MRI showing a large lobulated enhancing solid mass in the sellar and suprasellar regions (white arrows). The mass extends into the subfrontal lobes and in between the two frontal horns causing compression of foramen of Monroe. Inferiorly the mass extends to the sphenoid sinus and into the nasal cavity. The mass appears to encase the cavernous sinus and the optic chiasma.

**Figure 2:**
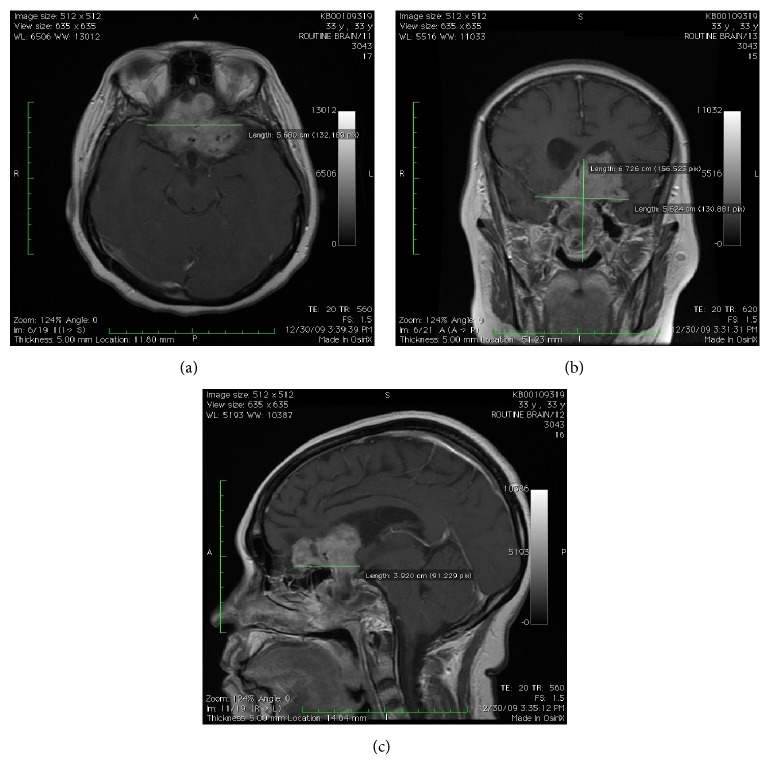
Contrast enhanced axial (a), coronal (b), and sagittal (c) T1-weighted sequence on MRI at 12-month follow-up showing reduction in size of the pituitary macroadenoma following treatment with cabergoline.

As the patient refused any surgical intervention, she was discharged home with bromocriptine 2.5 mg once daily with subsequent uptitration to twice daily and thyroxine. She was extensively counselled regarding the risk of worsening CSF leakage after DA treatment and the risk of meningitis. Nevertheless she was not started on any prophylaxis antibiotic. The patient was followed up for about a year and, within this period, serial serum prolactin and magnetic resonance imaging (MRI) of pituitary gland were performed to monitor the disease progression (refer to Table 1). On baseline MRI, a large lobulated enhancing solid mass was seen in the sellar and suprasellar regions extending into the subfrontal lobes and in between the two frontal horns causing compression of foramen of Monroe (refer Figure 1). Inferiorly, there was extension of the mass to the sphenoid sinus and into the nasal cavity. The mass appeared to encase the cavernous sinus and the optic chiasma. On follow-up, she did not tolerate bromocriptine well and hence changed to cabergoline 0.5 mg per week (titrated accordingly). There was clinical improvement seen with cessation of CSF leak, marked reduction of serum prolactin (serum prolactin dilutional assay of 14,734 mIU/L at 12 months of treatment), and radiological evidence of tumor shrinkage on MRI (refer Figure 2). Throughout the follow-up, there were no symptoms and signs to indicate meningitis. Although she claimed that her symptoms improved with treatment; however, her visual status remained the same and her menstruation did not commence.

## 3. Discussion

This report illustrates an unusual case of late presentation of giant macroprolactinoma with CSF leaks. The occurrence of rhinorrhea as the presenting symptom in treatment-naive prolactinomas is an extremely rare situation. In 2012, Lam et al. characterized the clinical scenarios most closely associated with spontaneous and medically induced CSF leaks in 52 patients with pituitary adenomas identified from 29 articles (published from 1980 through 2011) [5]. A total of 38 patients developed CSF rhinorrhea following initiation of medical therapy, whereas spontaneous CSF leakage developed as the presenting symptom in 14 patients. Forty-two patients (81%) had prolactinomas, with the remaining patients having other tumors such as nonfunctioning pituitary adenoma and growth hormone-secreting adenoma. Infrasellar tumor invasion into the paranasal sinuses was specifically reported in 56% of patients. The pharmacotherapy associated with CSF leakage was DA (97%) and somatostatin analogs (3%). Nonsurgical management was successful in four patients while 46 patients (88%) underwent surgical intervention to treat the CSF leak and/or resect the tumor. In our patient, CSF leakage appeared to be a result of direct extension of tumor through diaphragma sella and into the sphenoid sinus. Fager has suggested that the tumor may function as a ‘stopper' and once necrosis from infarction or hemorrhage occur, the lesion will no longer be able to block CSF flow and leakage could occur such as the situation after treatment with medical or radiation therapy [11]. On the other hand, erosion of the skull floor by pituitary adenomas may not necessarily cause CSF rhinorrhea, but alteration in CSF dynamics and pressures may play a role whereby pituitary tumor would generate intracranial hypertension which would be relieved by CSF leakage through an anatomically fragile area in the base of the skull.

In managing the patient, interpretation of prolactin level must be made with complete clinical scenario and imaging correlation to avoid diagnostic pitfalls which may lead to mismanagement. In this case, in the context of huge macroprolactinoma, the prolactin level of 4200 mIU/L was considered disproportionately low as previous study had shown correlation between prolactinoma volume and serum prolactin level. Therefore, dilutional assay was performed to avoid ‘high-dose hook effect', revealing true serum prolactin of 250,688 mIU/L. An extremely high level of prolactin may interfere with the assay and produce low readings. Since there is not enough antibody to bind to both ends of all antigenic prolactin peptides and most of the prolactin is now complexed to a single antibody, only few remaining prolactin peptides are detectable resulting in a falsely low value. Therefore, there is a proportional increase in assay titers up to a certain level as the antigen concentrations increase. Antigen concentrations above this threshold level would ‘hook' down the assay values and subsequently cause very low measurements [12, 13]. After three months of bromocriptine treatment, repeated serum prolactin in this patient dropped markedly to 37,000 mIU/L. Our patient was clinically well but admitted to reducing the dose of bromocriptine to 2.5 mg once daily on her own. However, repeated MRI pituitary surprisingly showed no changes in tumor size. Looking back at the initial level of prolactin which is extremely high and the subsequent markedly reduced serum prolactin level without radiological improvement should raise the possibility of concurrent presence of macroprolactinemia.

As patient could not tolerate bromocriptine intake, cabergoline was started. We observed further decrement in serum prolactin level and tumor size with improvement in treatment tolerability and compliance. There were no symptoms and signs to indicate meningitis in this patient during follow-up; hence, antibiotic prophylaxis was not warranted as long term use increases the emergence of bacterial resistance. Limited high-powered study and strong evidence due to rarity of this condition posed a great challenge to us in managing this patient. In previous studies, surgeons will emphasize surgery as the first line treatment for macroprolactinoma with spontaneous CSF rhinorrhea as it reduces the risk of ascending meningitis [5, 8, 9, 14, 15]. Nevertheless, in this case, patient had been having this symptom for many years without any episode of meningitis in the past. Therefore it was difficult to convince her that the risk of meningitis is real and surgical intervention will reduce the risk of this potentially fatal complication. Although counter-intuitive, the cessation of CSF leakage on continued DA therapy has been reported in isolated reports [9]. A reasonable hypothesis would be alteration in CSF pressure dynamics reducing the raised intracranial pressure resulting from large prolactinomas and allowing spontaneous healing of the CSF passage. Therefore, contrary to previous reports, surgical therapy may not always be necessary, and the use of DA does not necessarily cause worsening of CSF leakage and instead may produce spontaneous resolution as in this case.

## 4. Conclusion

As the patient strictly refused surgical option, this case demonstrated the effect of medical therapy alone in the setting of treatment-naive giant macroprolactinoma complicated by CSF leakage. There was marked reduction of serum prolactin level and radiological evidence of tumor shrinkage on MRI. Throughout her one-year follow-up, the use of DA alone has led to cessation of CSF rhinorrhoea and there was no clinical evidence of ascending meningitis. However her amenorrhea and visual defect remained unchanged.

## Figures and Tables

**Table 1:** Summary of disease progression with treatment.

Treatment Period	Serum Prolactin (mIU/L) (dilutional assay)	MRI Pituitary (tumor size)
Baseline	250,688	5.7 cm (AP) x 6.7 cm (W) x 7.5 cm (CC)
2 months	37,487	Essentially unchanged in size from baseline MRI
5 months	19,118	-
12 months	14,734	3.9 cm (AP) x 5.6 cm (W) x 6.7 cm (CC)

*∗*AP: anteroposterior, W: width, CC: craniocaudal.
